# Advances in real-time object tracking: Extensions for robust object tracking with a Monte Carlo particle filter

**DOI:** 10.1007/s11554-013-0388-4

**Published:** 2013-12-20

**Authors:** Thomas Mörwald, Johann Prankl, Michael Zillich, Markus Vincze

**Affiliations:** Vienna University of Technology, Gusshausstr. 25–29, 1040 Vienna, Austria

**Keywords:** Tracking, Detection, Modelling, Pose estimation, Robotic perception

## Abstract

The huge amount of literature on real-time object tracking continuously reports good results with respect to accuracy and robustness. However, when it comes to the applicability of these approaches to real-world problems, often no clear statements about the tracking situation can be made. This paper addresses this issue and relies on three novel extensions to Monte Carlo particle filtering. The first, *confidence dependent variation*, together with the second, *iterative particle filtering*, leads to faster convergence and a more accurate pose estimation. The third, *fixed particle poses* removes jitter and ensures convergence. These extensions significantly increase robustness and accuracy, and further provide a basis for an algorithm we found to be essential for tracking systems performing in the real world: *tracking state detection*. Relying on the extensions above, it reports qualitative states of tracking as follows. *Convergence* indicates if the pose has already been found. *Quality* gives a statement about the confidence of the currently tracked pose. *Loss* detects when the algorithm fails. *Occlusion* determines the degree of occlusion if only parts of the object are visible. Building on tracking state detection, a *model completeness* scheme is proposed as a measure of which views of the object have already been learned and which areas require further inspection. To the best of our knowledge, this is the first tracking system that explicitly addresses the issue of estimating the tracking state. Our open-source framework is available online, serving as an easy-access interface for usage in practice.

**Electronic supplementary material:**

The online version of this article (doi:10.1007/s11554-013-0388-4) contains supplementary material, which is available to authorized users.

## Introduction

This work is placed in the field of visual, model-based object tracking. It performs in real-time and is formulated as full 6 degree-of-freedom (DOF) pose estimation problem. Given the colour information of commonly available cameras, the task is to find the position and orientation (pose) of an object in space. To this end, the projection of a geometric model (i.e. triangle mesh), optionally together with texture information, is compared to the current image (frame). This comparison yields a measure, which is minimised with respect to the pose by applying a Monte Carlo particle filter (MCPF).

For a sequence of images, the trajectory of an object is observed, which is useful for various applications in the field of robotics, computer vision, augmented reality, surveillance, and so forth. In this work, we focus on autonomous robotics for several reasons. First, it requires real-time performance. Second, it relies on robust algorithms or statements about the current state of tracking. Third, it allows to test the tracker for real-world applications with all its difficulties and requirements. The goal is to provide a robot with all the information required to perform within real-world scenarios, such as grasping, object detection, tracking, learning physical object behaviour and so forth.

Another requirement in robotics is computational efficiency to react to observed situations in time. Consider a grasping scenario, where we want to use visual servoing to adapt the grasping movement on-line. Hence, we require real-time performance, i.e. processing time within the frame rate of a typical camera (25–50 Hz). Presently, we are using RGB data only, since we do not want to depend on sensors that also provide additional information such as depth as they might not be available for the user of our framework.

To meet all these requirements, we propose to tackle the core problem of detecting tracking failure and take advantage of supervisory knowledge to achieve automatic object tracking using texture mapping, pose recovery and online learning. Hence, the approach is based on the following methods:

- *Tracking-state-detection* (*TSD*) To know whether we are tracking correctly, whether the object is occluded or whether we lost track we employ our novel TSD method. The knowledge of the tracking state, including speed and confidence of tracking, allows for triggering online learning or pose recovery.
- *Texture mapping* We take advantage of texture, if available, to boost robustness of tracking, especially in cluttered scenes.
- *Pose recovery* To initialise tracking and recover lost tracks, we use distinctive features placed on the surface of the object model.
- *Online learning* We learn these feature points and surface texture of the object automatically while tracking.
- *Model completeness* A probabilistic formulation allows to reason if sufficient information of the object has been gathered.

The paper proceeds as follows. Section 2 gives an overview of related work on visual tracking algorithms. In Sect. 3, we formulate tracking as particle filtering using a modified version of the Bootstrap filter by [10] and show how to draw observations by projecting the model into image space. Section 4 introduces TSD which allows to reason about the current tracking quality, convergence and whether tracking has lost the object or is occluded. We show how surface texture and scale-invariant feature transform (SIFT) points, introduced by [19], of a tracked object can be learned online and how they are used for re-detection. In Sect. 5, we evaluate our approach with respect to the requirements established above and in Sect. 6 we conclude and discuss the methods proposed.

## Related work

For a robot operating in a complex unpredictable environment, the challenge is to develop a tracking method that is robust to different lighting conditions, partial occlusion, and motion blur. Today, this is achieved best by model-based tracking of objects and numerous solutions using different feature types, models and mathematical frameworks have been developed, where today's computational power allows for several real-time solutions. However, practical application of these methods is often limited for various reasons. For example, some methods report good results, without giving actual numbers on accuracy, such as [1, 14, 21, 22]. Approaches described by [21, 23, 34, 35] are capable of handling partial occlusion or changing lighting conditions but cannot differentiate between deteriorating tracking conditions and lost tracks. Some methods are restricted in their degrees of freedom, e.g. 2.4 radians of rotation as suggested by [23], require off-line learning (e.g. [34]) or are limited to either textured (e.g. [28, 33 ]) or low-textured objects (e.g. [36]). Also recovery from lost tracks is rarely handled with a few exceptions given by [28, 33], which are tracking-by-detection approaches.

Recently, the results reported by tracking-by-detection approaches are quite promising, especially with respect to speed. In the work of [11, 29] templates are exploited to achieve real-time performance. Using information about the 3D shape and taking advantage of depth sensing increase robustness as stated in [6, 12, 17, 33]. Local patches lead to a sparse representation of the model and allow for pose estimation without any prior pose. For initialisation and re-detection, we exploit this property using SIFT. To track and verify the pose by the TSD, we use a dense representation, in particular, a textured 3D CAD model. The appearance information is encoded in the colour map embedded in the domain of the object surface. This allows us to robustly identify the respective tracking states (e.g. occlusion). However, it would be interesting to see a method similar to TSD for approaches based on template matching.

Also use edges and textures for tracking [21]. Their approach extracts point features from surface texture and uses them, together with edges, to calculate the object pose. This turns out to be very fast as well as robust against occlusion. Our approach not only uses patches but the whole texture, which usually lets the pose converge very quickly to the accurate pose. Since the algorithm runs on the GPU, it is as fast as the method by [21]. The work presented by [36] uses edge features to track but does not take into account texture information. This makes it less robust against occlusion. Since the search area in that approach is very small, it is also less robust against fast movement and gets caught in local minima.

Other approaches aim to solve most of the problems of tracking, such as [35] where the authors are matching the camera image with pre-trained key-frames and then minimizing the squared distance of feature points taking into account neighbouring frames. The approach described by [23] uses a modified version of the Active Appearance Model which allows for partial and self occlusion of the objects and for high accuracy and precision. Minimize the optical flow resulting from the projection of a textured model and the camera image [31]. To compensate for shadows and changing lighting, they apply an illumination normalisation technique.

In [16], the authors introduce real-time tracking to robotic manipulation. They use the method proposed in [20], where they project the CAD model into image space, and try to minimize a cost functional for the distance to image edges found along the gradients of the edges of the model. The work presented in [8] describes an approach for real-time visual servoing using a binocular camera setup to estimate the pose by triangulating a set of feature points. Similar to our approach, Sánchez et al. [33] take advantage of robust Monte Carlo particle filtering to determine the pose of the camera with respect to SIFT features, which are localised in 3D using epipolar geometry.

Eextend visual tracking with a particle filtering by an initialisation based on key-point correspondences in a RANSAC scheme [2]. For re-initialisation, they propose to identify lost tracks by the efficient number of particles as given by [4], which we also use in our work.

**Fig. 1:**
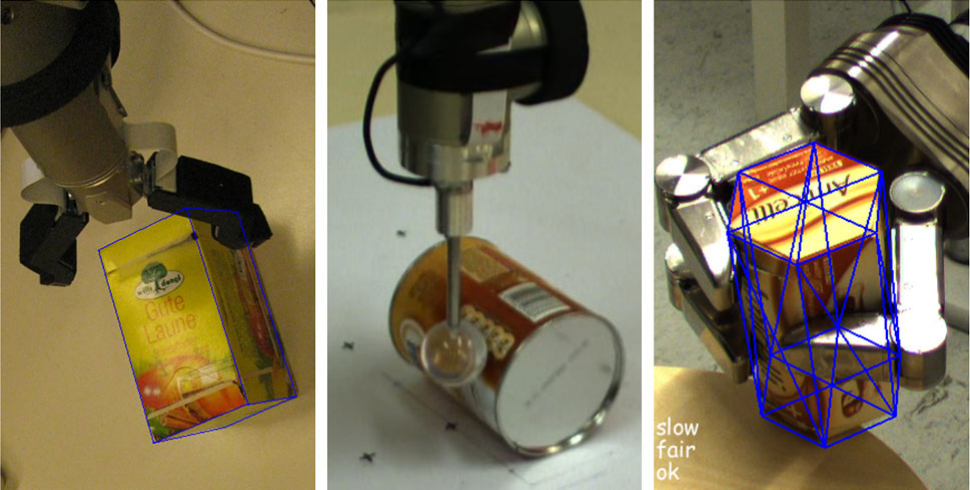
Tracking for robotic applications.* Left* grasping;* middle* learning about object motion;* right* grasp stability

Our approach builds on the work of [25, 37], and extends and improves the methods given by [27] (Fig. 1).

## Pose estimation

The full 6 DOF pose of the object is identified using colour and edge information from shape and texture. We project a model, typically consisting of triangles or quads with attached texture, into image space and compare it with the camera image. The pose is estimated using a modified version of the sequential importance resampling (SIR) particle filter as detailed by [5]. Image processing methods such as Gaussian smoothing and edge extraction as well as pixel-wise comparison of the projected model are accelerated using a typical graphics processing unit (GPU).

**Fig. 2:**
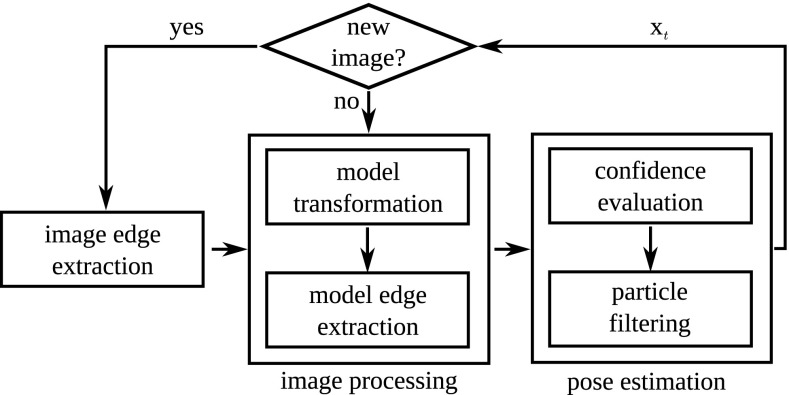
Tracking by iterative particle filtering. The pose is refined using our modified MCPF until a new image is provided by the camera. Together with the *confidence dependent variation*, this improves robustness and accuracy

Figure 2 shows our implementation of pose estimation. The pose is refined using iterative particle filtering until new data arrive from the image capturing pipeline. If this happens, the image edges are updated. Otherwise, the model is transformed according to the particles at frame *t* − 1. The edges of the model are extracted and matched with the current image edges. Subsequently, the weights are updated and the particles are re-sampled with replacement.

### Transformations on the *SO*(3)

Visual observation of the trajectory of the object is the problem of finding the transformations $\mathbf{{T}}_t$ given a sequence of images *I*_*t*_, sampled over the time. Since we constrain the tracking approach to rigid objects, the trajectory can be described as transformations on the rotation group *SO*(3). These are represented as

1 $${\mathbf{{T}}}({\mathbf{{x}}}) = \left[ \begin{array}{cc} {\mathbf{{R}}} & {\mathbf{{t}}} \\ {\mathbf{{0}}} & 1 \\ \end{array} \right],$$

where $\mathbf{{R}}({\varvec{{\theta}}})$ is a rotation matrix and $\mathbf{{t}} = [x, y, z]^T$ a translation, respectively. Rotations are realised using unit quaternions ${\mathfrak{q}}$ with ${||\mathfrak{q}||} = 1,$ which constrains $\mathbf{{R}}$ to be an element of the *SO*(3). They provide a simple way to represent uniform axis-angle rotations and avoid the gimbal lock which occurs when trying to model rotations by Euler angles. Quaternions are extensions to the complex numbers,

2 $${\mathfrak{q}} := r + \theta_x{\mathfrak{i}} + \theta_y{\mathfrak{j}} + \theta_z{\mathfrak{k}}$$

conveniently written as

3 $${\mathfrak{q}} := r + \boldsymbol{{\theta}}$$

${\mathfrak{i},\,\mathfrak{j},\,\mathfrak{k}}$ are imaginary units satisfying ${{\mathfrak{i}}^2 = {\mathfrak{j}}^2 = {\mathfrak{k}}^2 = {\mathfrak{ijk}} = -1.}$ A rotation by α radians about the axis $\mathbf{{u}}$ is defined as quaternion by

$${\mathfrak{q}} := \cos(\alpha/2) + {\mathbf{{u}}} \sin(\alpha/2)$$

Let $\mathbf{{a}}$ be an ordinary vector in ${\mathbb{R}^3}$ represented as quaternion with its real value *r* = 0, then a rotation of this vector is simply the quaternion product.

4 $$\tilde{{\mathbf{{a}}}} = {\mathfrak{q}}{\mathbf{{a}}}{\mathfrak{q}}^{-1}$$

Since the squared coefficients of unit quaternions sum up to 1, *r* is given by θ_*x*_, θ_*y*_ and θ_*z*_. Together with the translation $\mathbf{t},$ this results in a state vector of 6 DOF:

$${\mathbf{{x}}} = [x, y, z, \theta_x, \theta_y, \theta_z]^T$$

### Monte Carlo particle filtering (MCPF)

A particle filter, such as the sequential importance resampling (SIR) or the Bootstrap filter, explained in [4] and more detailed in [5], estimates the current state $\mathbf{{x}}_{t}$ based on the previous state $\mathbf{{x}}_{t-1}$ and the current observation $\mathbf{{y}}_{t}.$

5 $$\begin{aligned} {\mathbf{{x}}}_t &= f({\mathbf{{x}}}_{t-1},{\mathbf{{u}}}_{t-1}) + {\mathcal{N}}_{t-1}\\ {\mathbf{{y}}}_t &= g({\mathbf{{x}}}_t). \end{aligned}$$

We assume a static motion model, without taking into account external forces $\mathbf{{u}}_{t-1},$ yielding $f(\mathbf{{x}}_{t-1},\mathbf{{u}}_{t-1}) = \mathbf{{x}}_{t-1}.$ The observation $g(\mathbf{{x}}_t)$ is based on the image gradients and colour values.

**Fig. 3:**
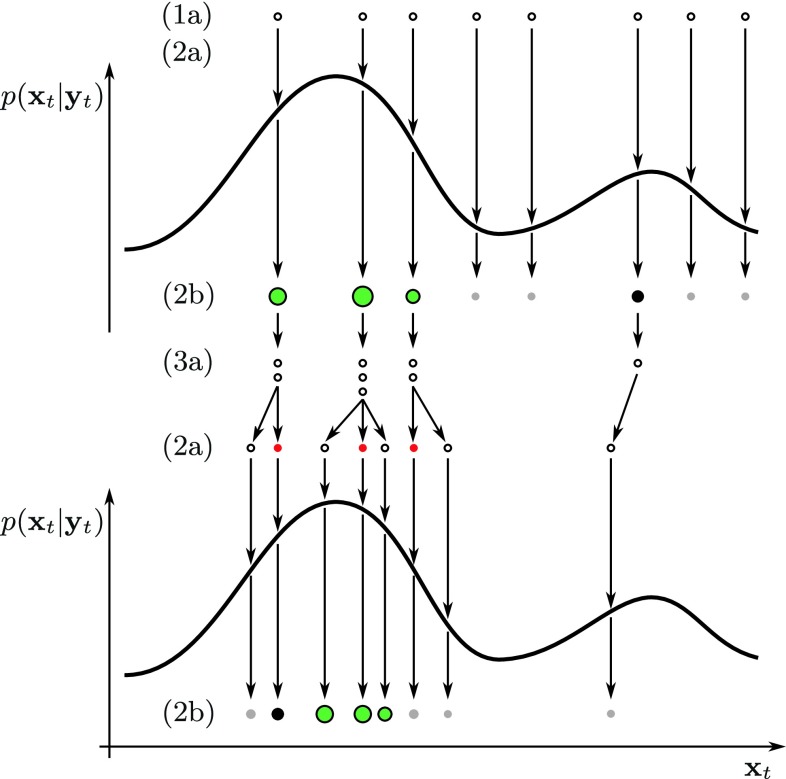
(*1a*) The classical MCPF starts with a uniformly weighted distribution of particles. (*2b*) The weight for each particle is evaluated, which results in an approximated distribution. (*3a*) According to the importance weights, the selection step assigns weak particles (*grey*) to the fittest (*green*). (*2a*) In our extension of the MCPF, all particles are perturbed using Gaussian noise except one, whose pose is fixed (*red*). Afterwards, the weights are evaluated again closing the loop. (The labels correspond to Algorithm 1.)

Figure 3 and Algorithm 1 show the behaviour of a Monte Carlo particle filter which sequentially resamples and replaces the particles depending on their weights. In the initial phase (1a), the particle distribution is initialised using a pose $\mathbf{{x}}_0$ given by user input or by a feature-based object detection system as described in Sect. 4.2. The particles are sampled from the normal distribution $\mathcal{N}(\mathbf{{x}}_0,{\varvec{{\sigma}}}_b).$ The confidence value *c*_0_ is set to 1. According to Eq. (7), this leads to an initial variance of ${\varvec{{\sigma}}}_0=0$ and, therefore, to no perturbation at all in step (2a). Note, that during tracking (i.e. *t* ≥ 1) the confidence value *c*_*t*_ is typically below 1. 
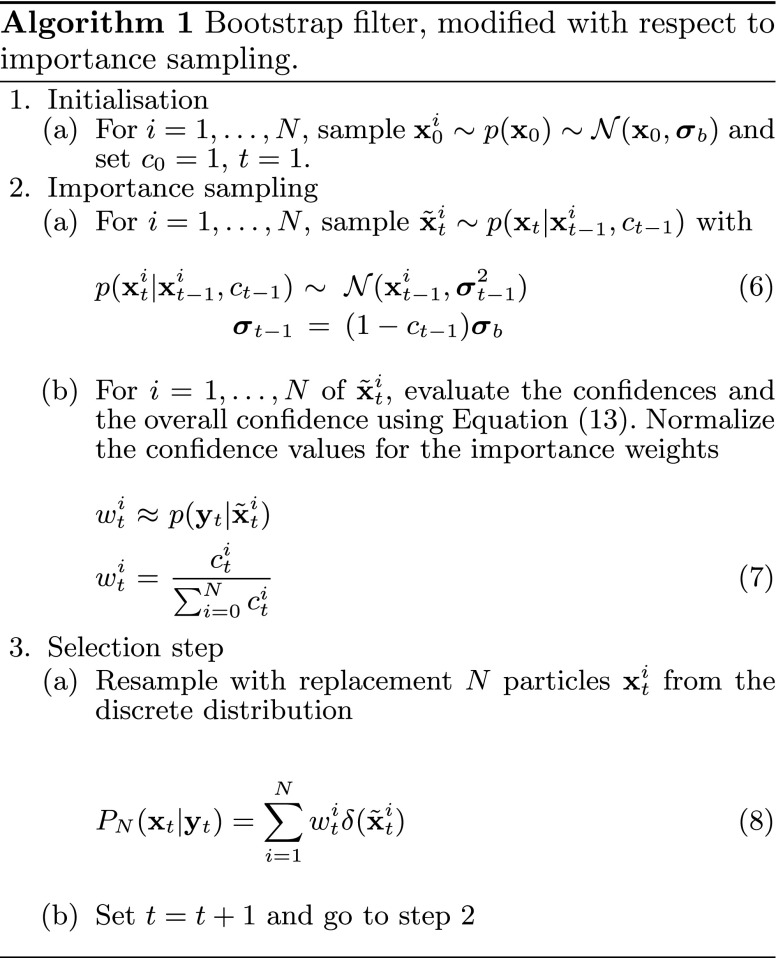

Given the observations ${\{\mathbf{{y}}_t|t\in\mathbb{N} \cup \{0\}\},}$ our aim is to estimate the posterior distribution $p(\mathbf{{x}}_t|\mathbf{{y}}_{t}).\,\mathbf{{y}}_t$ corresponds to the current image given by a camera sensor. In step (2b) for all poses $\mathbf{{x}}_t^i,$ the importance weights are evaluated, approximating the probability distribution of observations $p(\mathbf{{y}}_t|\mathbf{{x}}_t).$ The posterior distribution is given by the *Bayes’ theorem*.

The key idea of the MCPF lies in the approximation of $p(\mathbf{{x}}_t|\mathbf{{y}}_t)$ with a discrete distribution $P_N(\mathbf{{x}}_t|\mathbf{{y}}_t).$ Particles with low weights are eliminated, whereas the ones with high weights are multiplied. The final pose reported by the tracker is the weighted mean of the best $\overline{N}<N$ particles, $\overline{\mathbf{{x}}}_t.$ This is the classical , introduced by [10], which is typically applied for visual tracking as it has several advantages. First, it is very easy to implement. Second, the algorithm can be efficiently executed in parallel which we exploit using the GPU. And third, it is to a large extent modular which allows to replace certain steps by more sophisticated methods as follows.

*Confidence dependent variation* In the sampling step (2a) of Algorithm 1, we adjust the amount of system noise $\mathcal{N}$ according to the confidence of the previous tracking step *c*_*t*-1_. This means that as the confidence of the particles increases, their degree of distribution decreases, leading to faster convergence and less jitter. Given the requirements for tracking accuracy and speed for a typical table top scenario, we chose a basic standard deviation ${\varvec{{\sigma}}}_b = [\sigma_x,\sigma_y, \sigma_z, \sigma_{\theta_x}, \sigma_{\theta_y}, \sigma_{\theta_z}]^T$ with* σ*_*x*,*y*,*z*_ = 0.03 m for the translational and* σ*_θ_ = 0.5 rad for the rotational degrees of freedom.

**Fig. 4:**
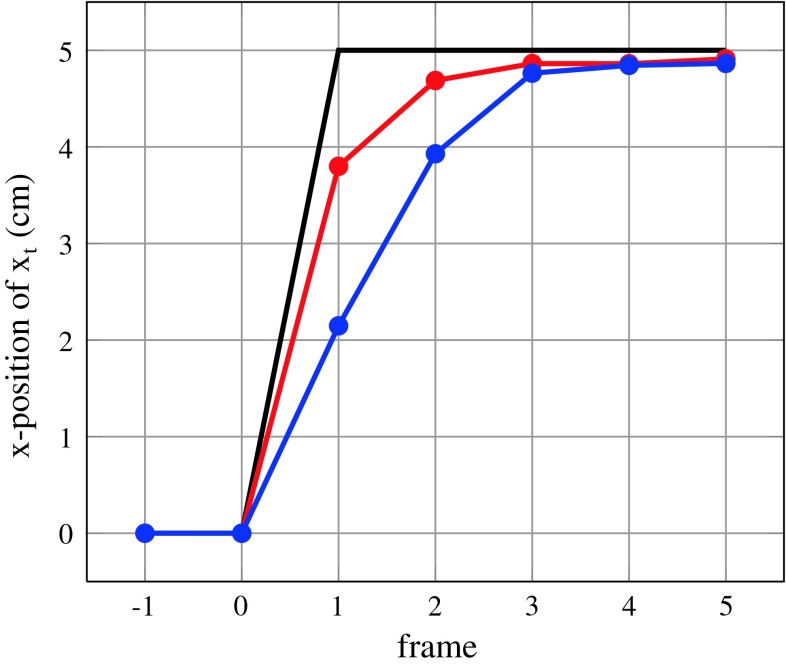
Step response showing the faster convergence of iterative- (*red*, 8 × 100 per frame) against conventional particle filtering (*blue*, 1 × 800 per frame). Both are using the same total number of particles

*Iterative particle filtering* As proposed in previous works by [24, 31], iterative particle filtering increases responsiveness to rapid pose changes. Therefore, steps 2 and 3 of Algorithm 1 are performed several times on the same image. This means that the poses of the particles are iteratively shifted to the peak of the distribution. In contrast to pure one-time re-weighting of the existing particles, this leads to a better approximation of the distribution $p(\mathbf{{x}}_t|\mathbf{{y}}_t)$ per image. Consider the situation where the time between two consecutive frames allows for evaluation of a total of 800 particles: Fig. 4 shows the improvement over conventional particle filtering when using 1 iteration with 800 particles versus 8 iterations with 100 particles. The latter, iterative version follows the object motion much faster.

**Fig. 5:**
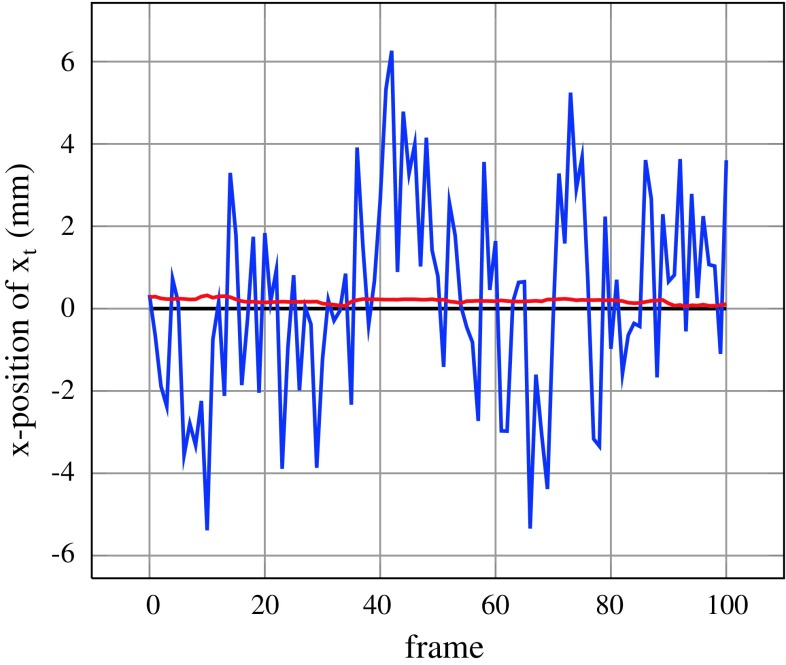
Visible jitter of the pose $\overline{\mathbf{{x}}}_t$ (*blue*) and improvement when fixing the pose of the best particles (*red*)

*Fixed particle poses* Since we want to perform in real-time, we use a limited number of particles which causes jitter of the final pose $\overline{\mathbf{{x}}}_t.$ At the same time, * σ*_*t*_ is never 0 (*c*_*t*_ < 1). This means that the best $\overline{N}$ particles will disperse around the true pose. With a sufficiently large number of particles, this would not be a problem, but due to our small number of particles it results in visible jitter. Instead of increasing the number of particles, sacrificing real-time performance, we use the following heuristic. The idea is to keep the pose of the best particles fixed instead of sampling from $\mathcal{N}.$ In detail, for each set of particles, with the same prior $\tilde{\mathbf{{x}}}_t^i,$ one is chosen where no noise is applied (Step 3a to 2a in Fig. 3). Obviously, this only makes sense if there are more than one particles in the set. The red particles in Fig. 3 indicate the set where the pose is fixed which we denote by $\mathbf{{X}}^f_t.$ This ensures convergence, efficiently reduces jitter and increases robustness of tracking as shown in Fig. 5.

### Image processing and confidence evaluation

At time-step *t* for each particle *i*, we project the model of the object into the image space using the transformation $\mathbf{{T}}^i.$ For simplicity, we skip *t* in the mathematical formulations since the following equations are computed in the same time-step. The geometry of the model is defined by vertices and faces. The texture, i.e. colour of the model is aligned to the faces by employing UV mapping, a standard technique of computer graphics. In image space, we compute the colour gradients of the model $\mathbf{{g}}^i_M$ and of the image captured by the camera $\mathbf{{g}}^i_I,$ where ${\mathbf{{g}} \in \mathbb{R}^2.}$ For each point (*u*, *v*) on the model *M* in image space, we can compute the difference between both of the gradients at that position, by superimposing the projected model over the image. The match *m*_*g*_^*i*^ of a particle is defined as the sum of the differences of the gradients, and *s*_*g*_^*i*^ is a normalising constant given by the sum over all model gradients.

9 $$\begin{aligned} m^i_g &= \sum\nolimits_{(u,v)\in{M}} |{\mathbf{{g}}}_{M}^i\left(u,v\right) - {\mathbf{{g}}}_{I}^i\left(u,v\right)|\\ s^i_g &= \sum\nolimits_{(u,v)\in{M}} |{\mathbf{{g}}}_{M}^i\left(u,v\right)| \end{aligned}$$

Additionally to the difference of gradients, the colour defined in hue, saturation, value (HSV) space is used for matching. Analogous to Eq. (9), the match for colour *m*_*h*_^*i*^ and its normalising constant *s*_*h*_^*i*^ are defined as

10 $$\begin{aligned} m^i_h &= \sum\nolimits_{(u,v)\in{M}} |h_{M}^i\left(u,v\right) - h_{I}^i\left(u,v\right)| \\ s^i_h &= \sum\nolimits_{(u,v)\in{M}} |h_{M}^i\left(u,v\right)| \end{aligned}$$

To achieve invariance with respect to brightness the hue values are used for matching the projected model *h*_*M*_^*i*^ and the image *h*_*I*_^*i*^. The advantage of using colour-based tracking is the increase of robustness against edge-based clutter. Of course it is less robust against changing lighting, but the combination of both kinds of cues can significantly improve the overall performance. The confidence of a particle $\mathbf{{x}}^i$ for matching gradients *c*_*g*_^*i*^ and colour *c*_*h*_^*i*^ is defined as

11 $$\begin{aligned} c^i_g &= \frac{1}{2}\left( \frac{m^i_g}{s^i_g} + \frac{m^i_g}{\frac{1}{N}\sum_{j=1}^N s^j_g}\right)\\ c^i_h &= \frac{1}{2}\left( \frac{m^i_h}{s^i_h} + \frac{m^i_h}{\frac{1}{N}\sum_{j=1}^N s^j_h}\right) \end{aligned}$$

where the first term is the match normalised with respect to *s*_*i*_. The second term is normalised with respect to the mean over all particles, de-weighting particles with a low number of pixels. This prevents the system from getting stuck in poses with a small number of pixels. The combined confidence of a particle is the product of the gradient- and colour confidence.

12 $$c^i = c^i_g c^i_h$$

The overall confidence of the current observation *t* is defined by the mean of the confidences of all particles *i* in the distribution.

13 $$c_t = \frac{1}{N} \sum_{i=1}^N c^i$$

## Tracking-state-detection (TSD)

Starting from a purely geometric representation of the object to track, robustness is improved by adding colour texture and feature-based information. Considering a cognitive robotic scenario, with as little user-input as possible, the key to automatically update the object representation is to detect the current state of tracking. This allows to identify good views for updating and improving the model representation. Furthermore, a quantitative measure of completeness of the model is necessary to determine views that have not been learned so far or where enough information is already available.

Observing the current state of the tracker is important for assessing the validity of the output as well as allowing to trigger recovery from lost tracks. TSD is a mechanism that indicates convergence, quality and overall state. It requires to reliably detect, whether the object is moving or the algorithm converged. For learning object detectors or classifiers, it might be necessary to know if a good view has been reached, or the object is occluded. But most important TSD has to distinguish between correct tracking, tracking failure or if the algorithm got caught in a local maximum. Therefore, TSD not only allows for learning about the object, but is also beneficial for tasks like pose recovery, robotic manipulation, visual servoing, learning physical behaviour from visual observations and so forth.

*Convergence rate* The convergence rate is important to determine if the object is moving or still. This measure must be independent from the quality of the current observations which might be influenced by occlusion, lighting or sensor noise. This means that just looking at the confidence value *c*_*t*_ is not enough. Observing the speed of the trajectory is not satisfying for three reasons. First, the first derivative of the position amplifies noise. Second, it depends on the size of the object and the point of view. And third, the elements of the speed vector derived from the position vector $\mathbf{{x}}_t$ are not of the same scale (translations versus rotations). Instead the fixed particles described in Sect. 3.2 are analysed. In more detail, the intersection and union of the set of fixed particles $\mathbf{{X}}^f$ at frame *t* and *t* − 1 are computed.

14 $$\begin{aligned} \hat{{\mathbf{{X}}}}^f &= {\mathbf{{X}}}^f_t \cap {\mathbf{{X}}}^f_{t-1}\\ \check{{\mathbf{{X}}}}^f &= {\mathbf{{X}}}^f_t \cup {\mathbf{{X}}}^f_{t-1} \end{aligned}$$

The intersection represents the particles that were not perturbed from one frame to the other. Then, the mean of the weights of the particles in $\hat{\mathbf{{X}}}^f$ normalised with respect to the weights of the particles in $\check{\mathbf{{X}}}^f$ is an indicator of convergence.

15 $$\begin{aligned} &v = \frac{1}{\sum_{j=1}^{\check{N}}w^j} \sum_{i=1}^{\hat{N}} w^i \\ & \quad \hbox{with }w^i({\mathbf{{x}}}^i | {\mathbf{{x}}}^i \in \hat{{\mathbf{{X}}}}^f)\\ & \quad \hbox{and } w^j({\mathbf{{x}}}^j | {\mathbf{{x}}}^j \in \check{{\mathbf{{X}}}}^f). \end{aligned}$$

**Fig. 6:**
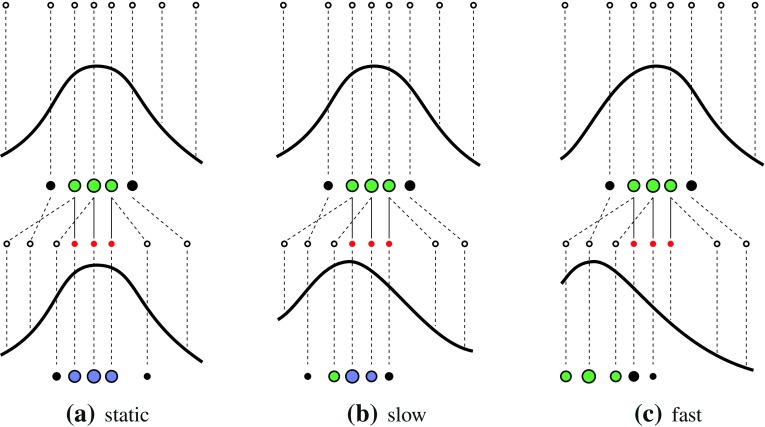
Convergence rate for a static (*left*), slow- (*middle*) and fast (*right*) moving distribution.* Green* particles are the fittest.* Red* ones are within the set of fixed particles $\mathbf{{X}}^f$ according to the definition in Sect. 3.2. * Blue* ones are within the set of intersection $\hat{\mathbf{{X}}}^f,$ from which the normalised mean weight is used for defining the convergence rate

Figure 6 illustrates convergence in the case of no (static), slow- and fast movement of the underlying distribution.

*Quality* To give a statement about the quality of the current pose, we use the overall confidence *c*_*t*_ which corresponds to the match of a pose hypothesis to the image evidence. We classify this measure to obtain qualitative statements by applying thresholds to distinguish if tracking is *good*, *fair* or *bad* (*c*_*t*_ > 0.5, 0.5 ≥ *c*_*t*_ ≥ 0.3 and *c*_*t*_ < 0.3, respectively).

*Loss* Another task of TSD is to determine if the algorithm is tracking the object correctly or has been lost and got stuck in a wrong local maximum of the probability distribution. For Monte Carlo methods, the effective particle size *N*_*eff*_ is introduced by [5]. Typically, it is approximated by

16 $$\hat{N}_{eff} = \frac{1}{\sum_{i=1}^N (w_t^i)^2}$$

leading to the definition of *loss* as

17 $$L := 1 - \hat{N}_{eff}/N$$

and pose recovery is triggered when *L* exceeds the threshold 0.5, i.e. when $\hat{N}_{eff} < N/2.$

**Fig. 7:**
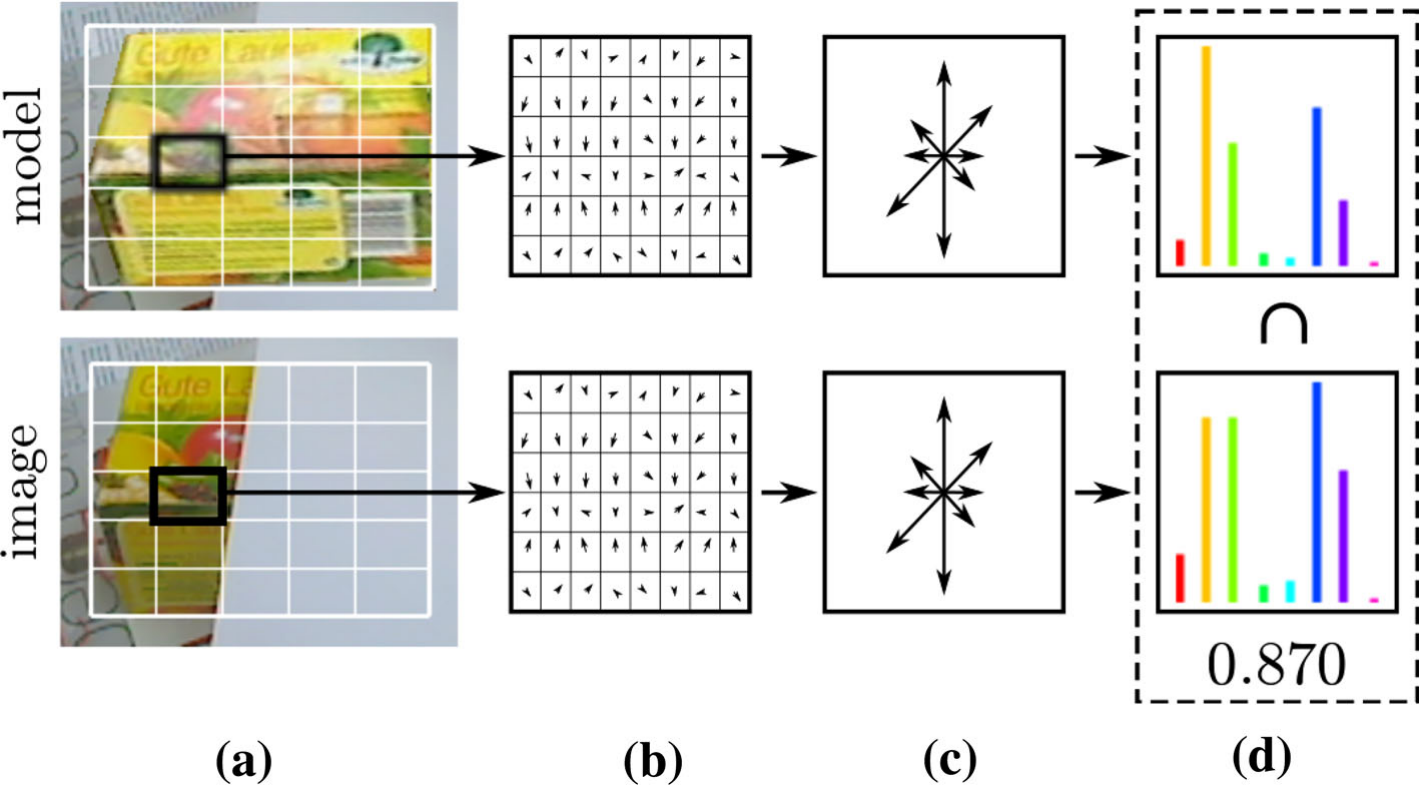
Histogram descriptor: the gradients and hue values of the subregions (** a**) are sampled (** b**) and accumulated into orientation histograms (**c**), both for the model and the image. The intersection of the histograms (**d**) represents the match of this specific subregion

**Fig. 8:**
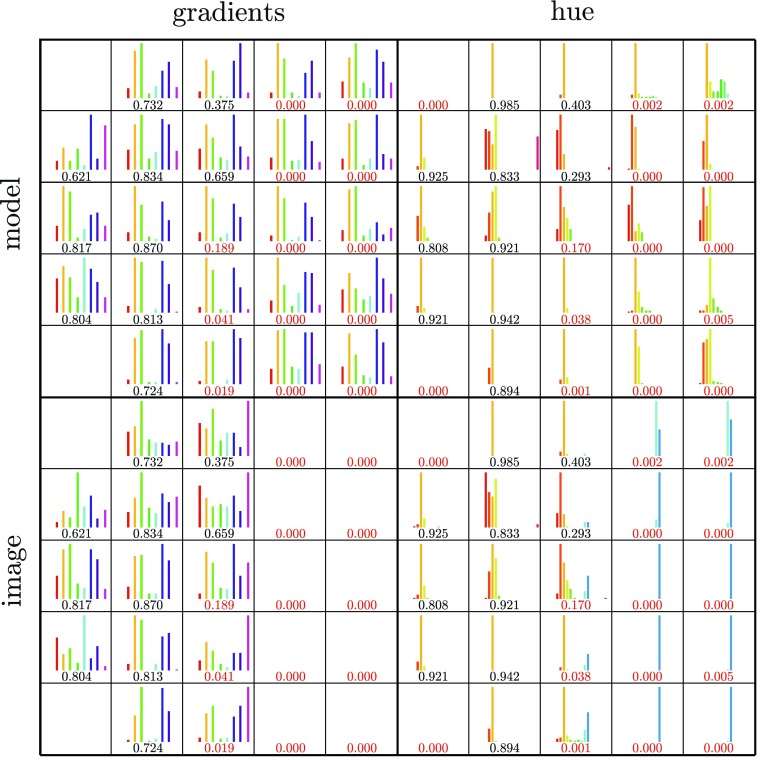
Histogram descriptor for the occluded object in Fig. 7. The intersection values of the gradients- and hue histograms approximate the amount and location of the occlusion

*Occlusion* A little more tricky is to observe whether the object is occluded or not. Therefore, a global histogram descriptor, taking into account edge- and colour information, is introduced. Similar to SIFT, gradients and hue values are sampled and accumulated into orientation histograms summarizing the contents over 5 × 5 partitions (Fig. 7). This is done for the camera image and the projection of the model. Figure 8 shows the histograms of all the subregions and their intersection values, respectively. This allows to determine how much of the object is occluded and which parts. Note that subregions which do not overlap the object sufficiently are not taken into account (e.g. top-left and lower-left subregion of Fig. 8).

### Texture mapping

Tracking is based on a CAD model which (initially) does not include surface texture. This is sufficient for non-textured objects, where all we can observe are edges resulting from occlusion and surface discontinuity. For textured objects, additional edges provided by the texture significantly improve robustness. The camera image provides the desired colour information of the object. The geometry of the object, i.e. the vertices, is projected into image space to determine their alignment with respect to the texture. TSD is employed to select good views. Further only faces of the model are taken into account, which are approximately pointing in the opposite direction of the camera view vector (i.e. faces that are parallel to the image plane). For those faces, the respective region of the camera image is cut out. The *u*, *v*-coordinates in pixel space are calculated by projecting the vertices using transformation $\mathbf{{T}}$ provided by the tracker and the camera intrinsics.

### SIFT mapping and object re-detection

While edges are well suited for fast tracking we use highly discriminating SIFT features for object detection (where again we use a GPU implementation [29]). Hence, we follow a standard approach similar to [3, 9] but our training phase differs in that we do not build a sparse 3D SIFT point model via bundle adjustment but use the 3D pose and object geometry already provided by the tracker. To this end, the view rays according to the *u*, *v* pixel coordinates of the SIFT points are calculated using the camera intrinsics. Then, the view rays are intersected with the faces of the object model at the current pose $\mathbf{{x}}_t$ to get the 3D positions with respect to the object pose. SIFT features falling outside the object boundary are discarded.

To speed up object detection, SIFT features are represented using a codebook (one per object). According to the codebook entry, each matched feature has several corresponding 3D model locations. To robustly estimate the 6D object pose, we use the OpenCV pose estimation procedure in a RANSAC scheme by [7], with a probabilistic termination criterion, where the number of iterations necessary to achieve a desired detection probability is derived from an estimate of the inlier ratio, which is taken to be the inlier ratio of the best hypothesis so far. So the number of RANSAC iterations is adapted to the difficulty of the current situation and accordingly easy cases quickly converge.

### Model completeness

Now that it is possible to learn texture and SIFT-based features of the model, the question arises when to stop learning. In other words, how much information is needed to represent the model sufficiently for tracking, initialisation and recovery of the pose. For tracking, completeness is achieved if the textures of all faces of the model are captured according to Sect. 4.1. Unfortunately, this cannot be applied to the SIFT-based model since detection suffers much more from angular deviation and scale. Therefore, Zillich et al. [37] propose a view-based probabilistic formulation indicating how likely it is to detect the object from a certain point of view. In more detail, the probability of detecting trained object view *o* (*o* = *true*), given object pose $\mathbf{{x}},$ is formulated using Bayes rule.

18 $$\begin{aligned} p(o|{\mathbf{{x}}}) &= \frac{p({\mathbf{{x}}}|o)p(o)}{p({\mathbf{{x}}})} = \frac{p({\mathbf{{x}}}|o)p(o)}{\sum_{k\in {\mathcal{O}}}p({\mathbf{{x}}}|o=k)}\\ o &\in {\mathcal{O}} = \{\text{true}, \text{false}\} \end{aligned}$$

The probability $p(\mathbf{{x}}|o),$ i.e. of observing a particular pose $\mathbf{{x}}$ for a detected or missed object view *o* is estimated from labelled training data. These data are obtained by transforming a virtual object model with 1,000 random rotations, 252 scales and varying levels of artificial noise and blur. The prior *p*(*o*) is the probability of detecting the object at all, which might come from contextual information, e.g. the probability of an object being in a certain room. For our experiments, *p*(*o*) is set to 1. To come to a measure of *model completeness* the probability of detection over all learned views is taken.

19 $$\hat{p}(o) = \sum_{{\mathbf{{x}}}} \max_j p(o_j|{\mathbf{{x}}})p({\mathbf{{x}}})$$

where $p(\mathbf{{x}})$ takes into account that certain views are less likely than others (such as the underside of an object). This representation allows a robotic system to identify lack of information and to take action to learn more views (e.g. repositioning, moving the object or the camera, etc.). E.g. in the work of [37], a gain-cost-scheme is applied to drive exploration.

**Fig. 9:**
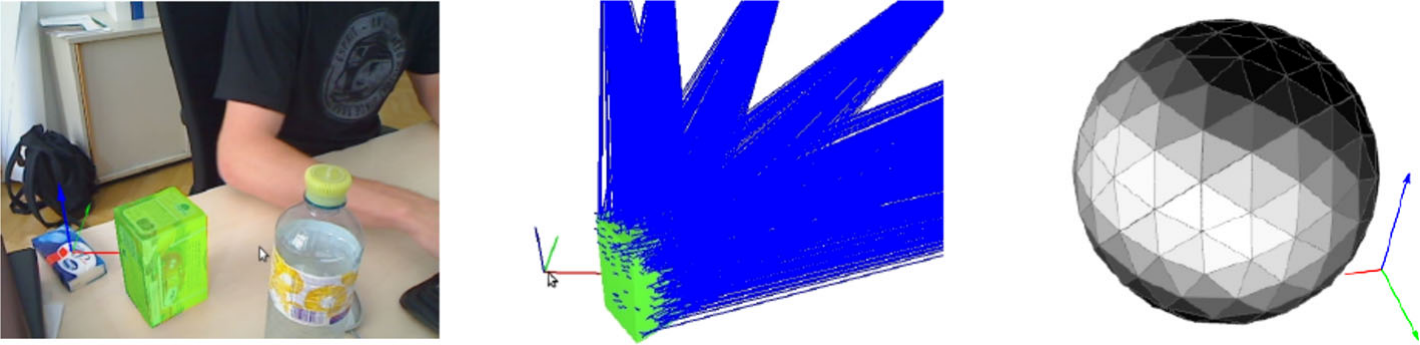
Model completeness. The object in the scene (*left*) and bundles of features with their view vectors (*middle*) after acquiring some views of the object. View sphere (*right*) with brighter shades of* grey* indicating that the object has been learned from the respective direction

In our approach, the object poses relative to the camera are represented by the unit sphere, disregarding the distance. Figure 9 shows such a sphere, where bright regions indicate viewing angles of high probabilities, whereas dark ones have not been learned so far.

## Results

We evaluated the approach using virtual rendered image sequences with known ground truth as well as live sequences where we obtain ground truth from a calibration pattern rigidly attached to the object. All experiments were performed on a PC with an Intel Core 2 Quad (Q6600, 2.4 GHz) CPU, a NVIDIA GeForce GTX 285 GPU and a Logitech Webcam Pro 9000 run at a resolution of 640 × 480 pixels.

### Evaluation of the tracking error

For a measure of the error, we used the scheme proposed in Sect. IV-B in [15], where a large number, $k=[1 \ldots\, K],$ of randomly chosen points ${\mathbf{{q}}^k \in \mathbb{R}^3}$ are rigidly attached to the object surface at the ground-truth pose and compared to the corresponding points ${\hat{\mathbf{{q}}}^k \in \mathbb{R}^3}$ of the tracked pose. The error at a specific frame *t* is then approximated by

20 $$e_t = \frac{1}{K} \sum_{k=1}^K{|\hat{{\mathbf{{q}}}}^k_t - {\mathbf{{q}}}^k_t|}$$

i.e. the error is given in terms of surface displacement which is a more meaningful measure than the pose difference. Before evaluating our method in terms of the above error metric, let us briefly consider the possible sources of errors in our system, such as errors from calibration, geometric modelling, image quantisation and finally the tracking algorithm itself. Concretely, we identify the following sources of errors:

- *Mechanical error* Positioning the calibration pattern rigidly on the object introduces a small unknown error which can safely be considered to be in sub-millimetre range.
- *Camera error* The pose of the calibration pattern is detected with a standard DLT algorithm, followed by a non-linear optimisation of the pose using the sparse bundle adjustment implementation by [18]. Further, the rolling shutter of the camera used introduces additional errors, which is negligible for our speeds.
- *Quantisation error* Depending on image resolution, a digital camera introduces a pixel quantisation error. In our evaluation, we use a resolution of 640 × 480 with a focal length of ∼500 in pixel-related units. This leads to an error of about 0.5–1.5 mm when tracking at a distance of 0.5–1.5 m parallel to the image plane. This error is even higher for the orthogonal direction, which shown in Table 1.
- *Modelling error* For modelling boxes and cylinders we measured the main dimensions of the respective objects. Arbitrary-shaped objects are modelled using an RGB-D sensing device, namely the Asus Xtion Pro Live and subsequent Poisson triangulation by [13]. To achieve real-time performance, we simplified the models leaving out small details, chamfers or slightly bulging cardboard surfaces. Unfortunately we do not have a measure for the *Modelling error* but for the basic shapes (i.e. boxes and cylinders), where correct modelling is simple, we assume this error to be negligible.
- *Texturing error* We found that textures added during the modelling phase do not always align properly. Manually capturing textures triggered by pressing a button incorporates less error than automatic capturing based on tracking-state-detection.
- *Tracking error* The failure of the tracker to accurately locate the local maximum, depending on the challenges posed by current viewing conditions.

**Table 1:** Accuracy in mm

Target	Static		Dynamic	
Object	*x*, *y*	*z*	*x*, *y*	*z*
Boxes (virt.)	0.4	2.3	1.5	5.6
Boxes (real)	2.0	5.5	2.6	7.7
Cylinders (virt.)	0.9	4.4	2.4	10.0
Cylinders (real)	3.0	16.5	3.9	21.9
Skull (virt.)	2.5	4.2	3.5	14.5
Skull (real)	3.7	6.2	4.6	15.4
Truck (virt.)	0.6	8.3	3.3	13.4
Truck (real)	1.5	10.5	4.5	16.1
Spray (virt.)	1.0	8.3	3.3	18.2
Spray (real)	1.2	10.2	4.4	23.9
Detergent (virt.)	0.9	6.5	1.7	11.4
Detergent (real)	1.0	7.5	2.1	15.9
Train (virt.)	0.5	2.8	1.4	5.6
Train (real)	2.0	7.0	2.5	8.8

### Accuracy and precision

**Fig. 10:**
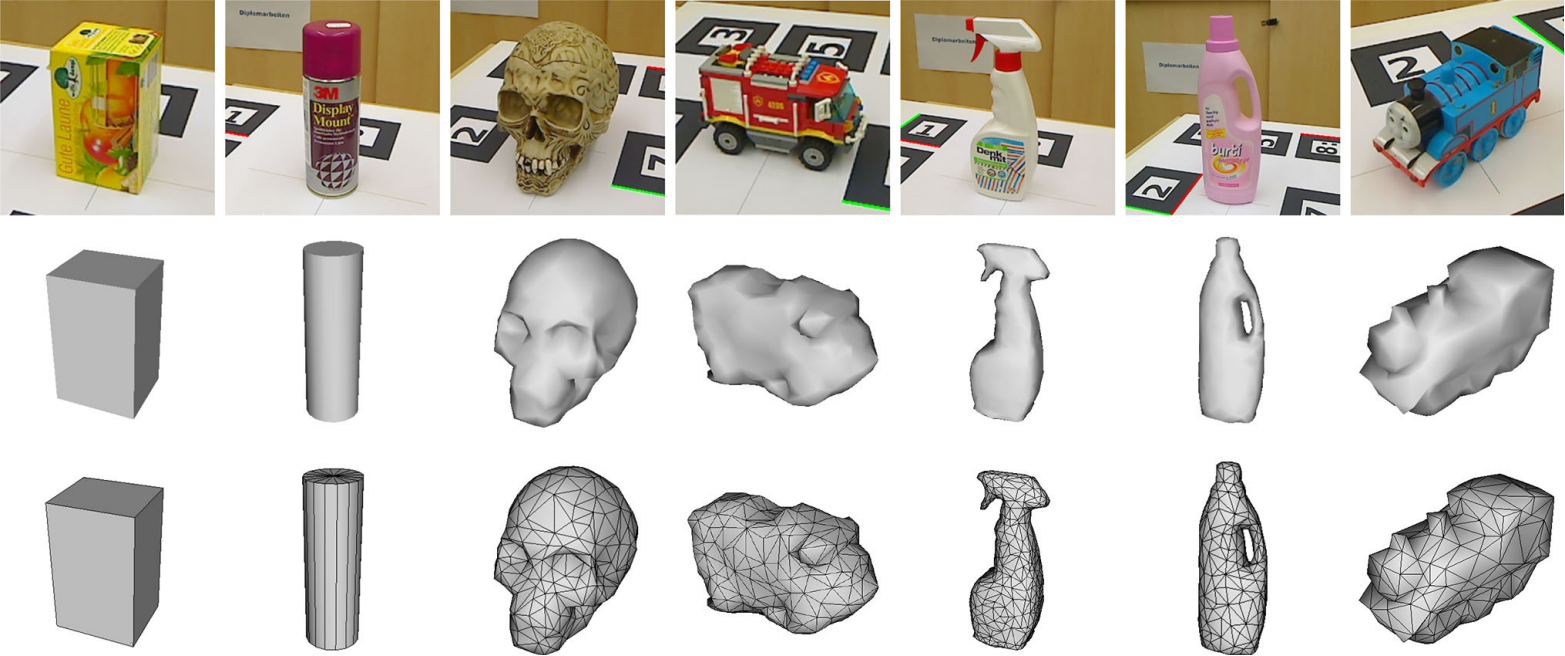
Objects used for evaluating accuracy, precision and performance. From* left* to* right*: box, cylinder, skull, truck, spray, detergent, train. The* bottom* rows show the untextured triangle meshes used for tracking

**Fig. 11:**
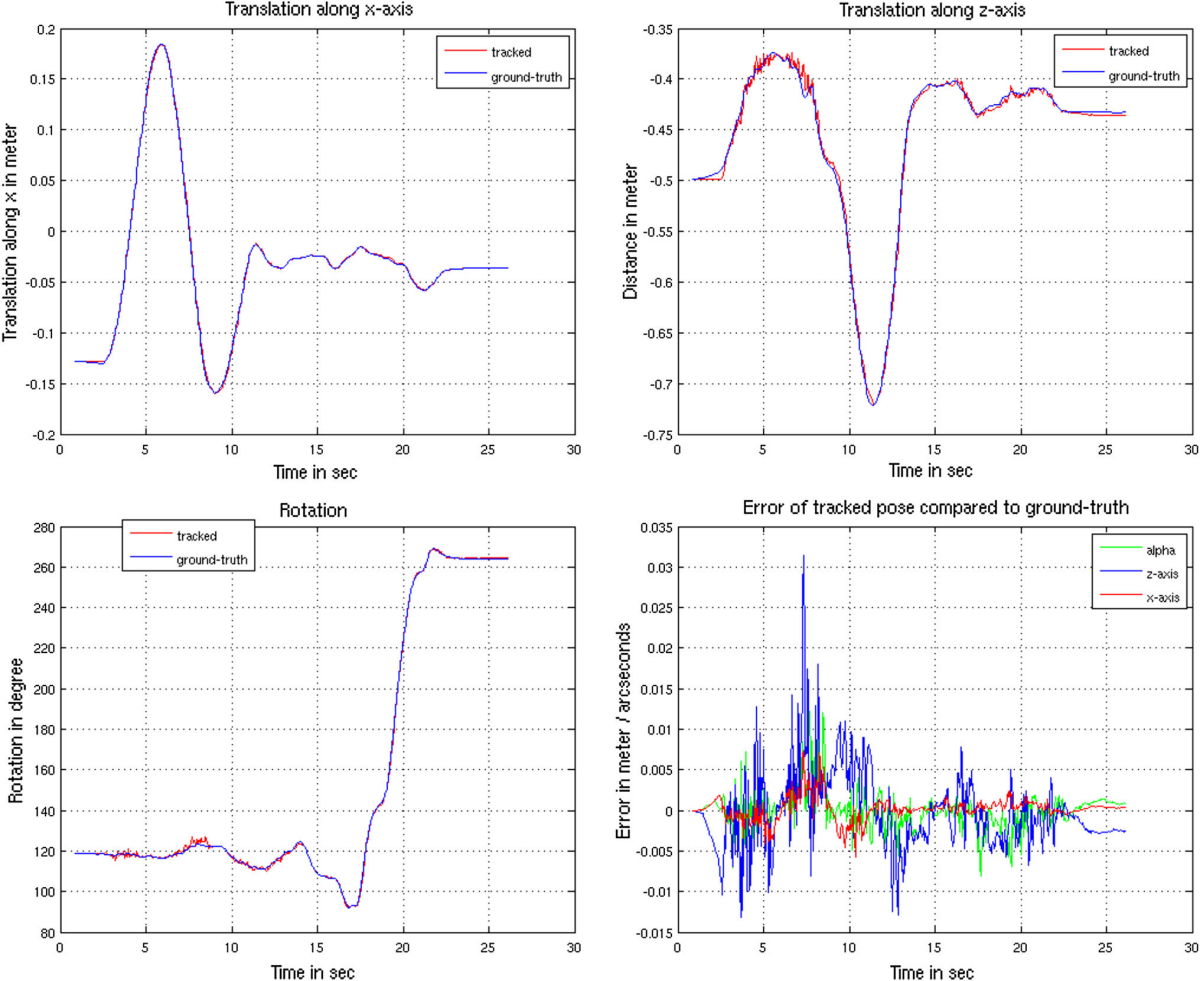
Trajectory of a tracked virtual object with 45 cm* x*-translation followed by a 70 cm* z*-translation and a rotation about the objects* y*-axis. The lower right figure shows the pose deviations, respectively

We evaluated accuracy and precision using 5 box shaped, 5 cylindrical and 5 arbitrary shaped objects depicted in Fig. 10 using sequences of 20–30 s length. An example trajectory is shown in Fig. 11.

Accuracy is defined to be the closeness of a quantity to its actual value, which in our case is measured using Eq. (20), where the pose of tracking is compared to the pose of the virtual object or the pose detected from the calibration pattern, respectively. We evaluated the mean accuracy with respect to the poses of several trajectories using

21 $$e = \frac{1}{Jt_e}\sum_{j=1}^J{ \sum_{t=1}^{t_e}{ e_{t} } },$$

where $j=[1\ldots\, J]$ are the trajectories of poses $t=[1\ldots \,t_e]$ under unchanged conditions, i.e. tracking *J* times on a sequence of *t*_*e*_ images.

Precision, also called repeatability, is the degree of deviation of a quantity under unchanged conditions, which is also measured using Eq. (20). For each frame *t*, the pose of tracking $\hat{\mathbf{{q}}}^k$ is compared to its own mean with respect to the number of repetitions *J*. i.e. the points of ground-truth $\mathbf{{q}}^k$ in Eq. (20) are substituted by

22 $${\mathbf{{q}}}^k_t = \frac{1}{J} \sum_{j=1}^J{\hat{{\mathbf{{q}}}}^k_t}$$

and precision is again given by Eq. (21).

Tables 1, 2 show the results of the accuracy and precision evaluation, where we evaluated two different cases: a *static* scene where we looked at the mean error of the pose after it converged within a few frames. And a *dynamic* scene where we observed the mean error of the trajectories. For evaluation, we used box shaped and cylindrical objects. The virtual objects show the *Tracking error* and *Quantisation error* (all other errors being ruled out), whereas the difference between virtual and real objects is due to *Mechanical*, *Camera*, *Modelling* and *Texture error*, where we assume the *Modelling* and *Texture error* to play the main roles. We evaluated the dynamic errors using trajectories including linear movement, rotation and their combination. Further, we considered real-world conditions like occlusion and changing illumination.

**Table 2:** Precision in mm

Target	Static		Dynamic	
Object	*x*, *y*	*z*	*x*, *y*	*z*
Boxes (virt.)	0.2	1.1	0.7	3.2
Boxes (real)	1.1	2.9	1.6	4.9
Cylinders (virt.)	0.4	1.9	1.3	5.7
Cylinders (real)	0.5	2.5	1.6	8.8
Skull (virt.)	1.3	2.9	1.2	4.8
Skull (real)	1.5	4.3	1.8	6.8
Truck (virt.)	0.3	2.1	2.2	5.6
Truck (real)	0.8	2.8	2.9	6.8
Spray (virt.)	0.4	2.0	1.0	4.5
Spray (real)	0.6	3.4	1.8	6.5
Detergent (virt.)	0.2	3.0	0.3	3.6
Detergent (real)	0.3	3.6	0.8	5.4
Train (virt.)	0.2	1.2	0.8	2.9
Train (real)	0.7	2.4	1.2	3.4

We can derive from Table 1 that curved objects are typically harder to track than box-shaped objects. A typical trajectory for arbitrary movement is shown in Fig. 11 where the tracked pose is compared to the virtual pose with respect to translations, rotations and the error measured by Eq. (20).

### Robustness

We tested our approach against various situations including fast movement introducing motion blur, occlusion, changes in lighting and large distances. Since robustness is hard to put in numbers the reader is referred to a video 1, to get an impression of how these various challenges are handled.

### Tracking-state-detection

**Fig. 12:**
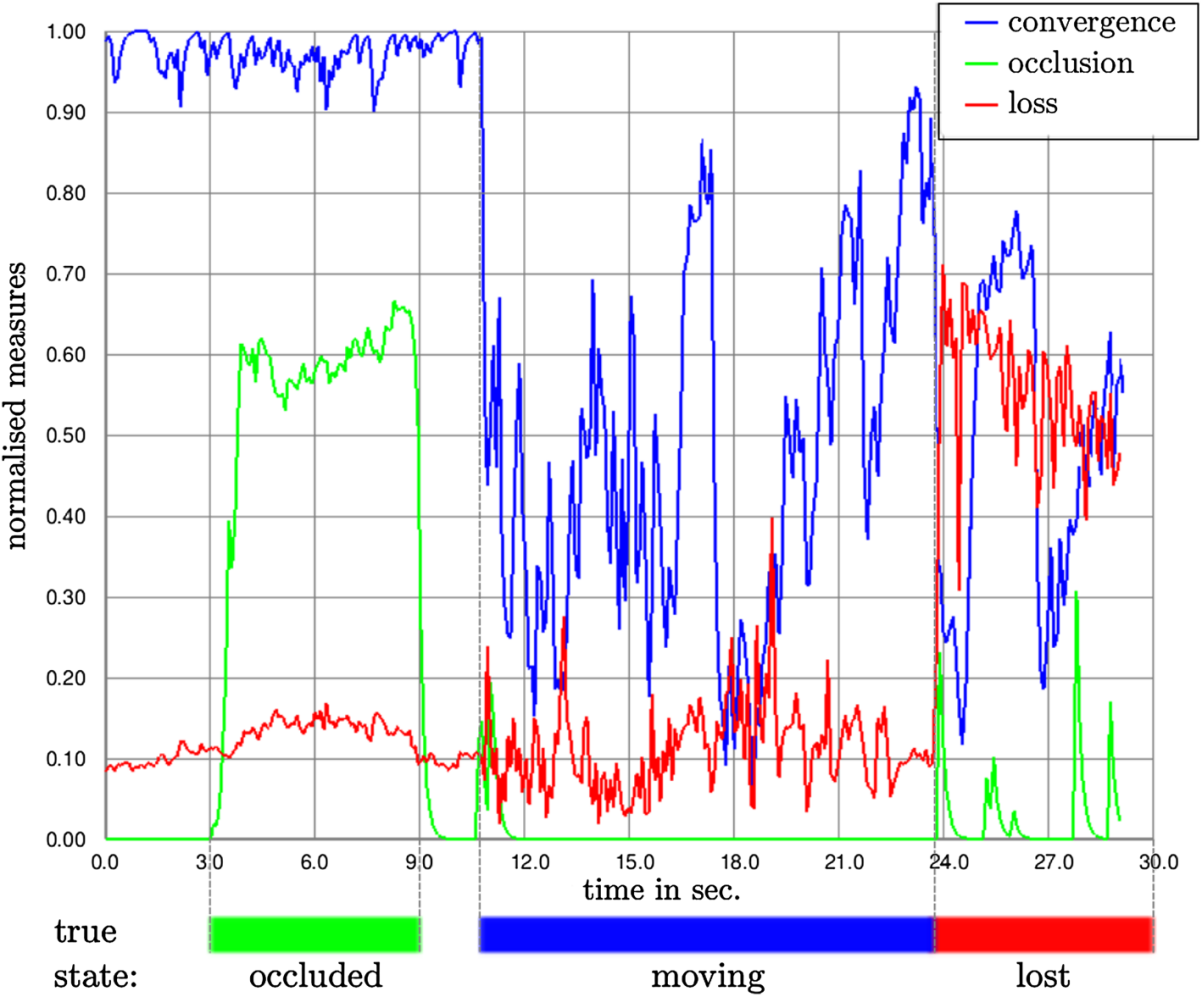
Output of the measures *convergence* (*blue*), *occlusion* (*green*) and *loss* (*green*) during tracking. Below the graph, the ground truth of the respective situation is highlighted in the corresponding colour

Figure 12 illustrates the different measures introduced in Sect. 4 compared to hand labelled ground truth. First, we partially occluded approximately half of the object by hand, resulting in an increase of the occlusion measure (3rd–9th second). At the 10th second, we started to move the object around leading to a decrease of convergence. Between the 23rd and 24th second, the object left the field of view, resulting in an immediate response of our loss detection.

### Performance

Processing time during tracking depends on the complexity of the model as well as on the number of particles used for tracking.

**Fig. 13:**
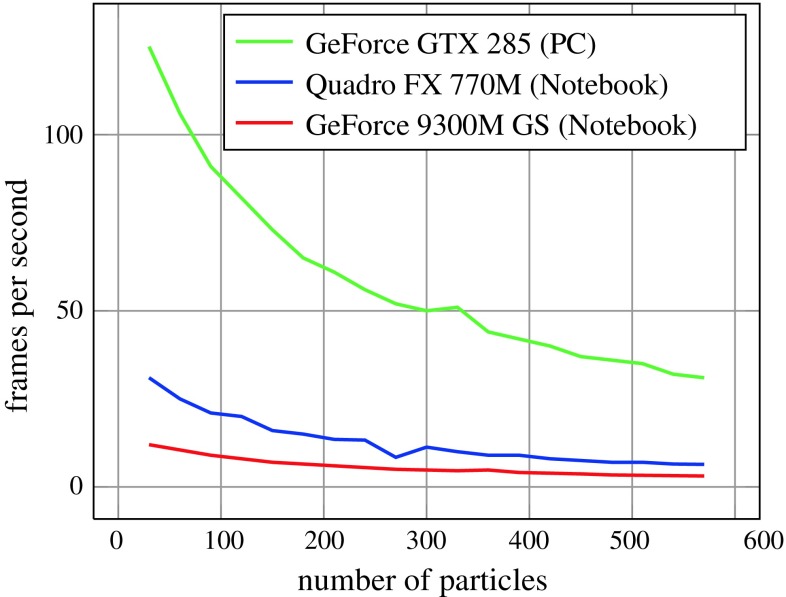
Frame rates with respect to the number of particles on different platforms for the *Box* model

Table 3 shows the frame rates for different numbers of faces and particles. 2 × 50, 3 × 100 and 4 × 300 indicates 2, 3 and 4 iterations using 50, 100 and 300 particles for each iteration, respectively. Figure 13 shows the frames per second on different GPUs with respect to the total number of particles used for tracking.

**Table 3:** Frame rates with respect to model complexity and number of particles

Number of faces	Frames per second		
	2 × 50	3 × 100	4 × 300
6	120	50	16
24	110	48	15
96	100	45	14
384	80	40	12
500	35	15	4
700	20	7	1

## Discussion

In this paper, we presented a robust method for model-based 6 DOF pose tracking. We have modified and improved state-of-the-art particle filtering approaches by various contributions. Defining the variance of the particles depending on their confidence from the previous observation yields faster convergence and less jitter. Fixing the pose of some of the best particles further reduces jitter as no good poses are lost due to re-sampling. Further, these fixed poses indicate the tracking state. Another improvement is the iterative structure of the particle filter leading to a faster convergence by sampling fewer particles more often.

Further, we have developed a method to determine the state of tracking. This allows us to reason about the quality of a certain trajectory and to identify good views. The first is useful, for example, when physical behaviour is learned by visual tracking, only taking into account trajectories of a certain quality as done by [15, 26]. The latter is used for learning texture and SIFT key points of certain views of the object.

A not so obvious but necessary requirement for TSD is detection of occluded objects as a special case of a tracking state. Its importance becomes clear when we want to update the existing information by the one given from a better view. Therefore, we need to know whether the object is occluded or not. This means that we have to detect views where such a situation occurs and mark them as being not good to learn from.

The methods presented in this paper provide a tool for use in robotic applications. Therein lies the main contribution to the community. Although a lot of tracking algorithms exists, there are hardly any that allow for robust tracking in harsh real world conditions and provide qualitative statements about the observations. Furthermore, our tracking framework is available for download. 2

## Electronic supplementary material

Below is the link to the electronic supplementary material.

MPG (19752 KB)

### Electronic supplementary material

Below is the link to the electronic supplementary material.

AVI (10676 KB)

## Footnotes

1
http://users.acin.tuwien.ac.at/tmoerwald/?site=5.

2
http://users.acin.tuwien.ac.at/tmoerwald/?site=4.
